# Development of the Korean Community Health Determinants Index (K-CHDI)

**DOI:** 10.1371/journal.pone.0240304

**Published:** 2020-10-08

**Authors:** Dun-Sol Go, Young-Eun Kim, Seok-Jun Yoon

**Affiliations:** 1 Department of Health Care Policy Research, Korea Institute for Health and Social Affairs, Sejong, South Korea; 2 Big Data Department, National Health Insurance Service, Wonju, South Korea; 3 Department of Preventive Medicine, Korea University College of Medicine, Seoul, South Korea; University of the Witwatersrand, SOUTH AFRICA

## Abstract

This study developed and validated a Korean community health determinants index (K-CHDI), which can be used to assess the health status of the community. To develop composite indicators, we followed the guidelines of the Joint Research Centre of the Organization for Economic Cooperation and Development. We reviewed previous studies and formed a theoretical framework to systematize our domains and indicators, which were decided through a Delphi survey of healthcare experts. Data on indicators were obtained from the Korean Statistics and Community Health Survey. We applied the Min-Max normalization method and measured weights by the analytic hierarchy process. Health outcomes were estimated using mortality, years of life lost, years lived with disability, and disability-adjusted life years by standardizing sex and age. The value of the index is between 0 and 1; higher values indicate more positive health determinants. K-CHDI for 250 subnational regions (cities, counties, and districts, or *Si·Gun·Gu*) were correlated with health outcomes. The correlation coefficient was stronger in large cities than in medium-sized areas and small areas, and the higher the K-CHDI group, the higher the coefficient. The K-CHDI represents a reference standard for estimating health status using health determinants as composite indicators at the subnational level.

## Introduction

In population health, various health determinants affect health gaps between groups, which is as important as the health gap between individuals [1]. Monitoring health differences and factors that may affect health variation multidimensionally with reliable, valid, and sustainable measurement is essential for planning related policies to eradicate those gaps. Most previous studies have evaluated health differences according to a single indicator, such as income or education. The composite index is composed of various indicators and calculated as a standardized value; it is highly useful for comparing health status between countries or regions.

The Global Burden of Disease (GBD) Study developed a socio-demographic index (SDI), suggesting that a country’s burden of disease varies according to its degree of socioeconomic development [2]. The SDI is a composite index of income per capita, educational attainment, and the total fertility rate of individuals under the age of 25. It has a geometric mean of 0 to 1, with higher values indicating a higher sociocultural level. However, in Korea, as the differences in health outcome by educational level and fertility rate are getting smaller, other health determinants have become important, such as employment status and resources. SDI has not enough factors that explain the Korean health determinants, thus, an index including health indicators to better explain the health differences among Koreans should be developed.

Our study aimed to develop a composite index to reflect the determinants of community health in Korea at the subnational level and to evaluate the validity and usability of the new index.

## Methods

### Study setting

The study included 250 Korean municipal-level administrative districts, comprised of 67 cities (*“Si”*), 114 counties (*“Gun”*), and 69 districts (*“Gu”*). The study was conducted in 2016, and used the most recent health outcomes data available.

The study protocol was approved by Korea University’s institutional review board (KUIRB-2018-0080-01).

### Development of a composite index for the community health determinants

For the construction of the Korean Community Health Determinants Index (K-CHDI), we followed the standard procedure described by OECD and JRC guidelines [3,4]. The steps were in order of choosing the theoretical framework, selection of indicators, standardization and aggregating indicators using weights.

#### Theoretical framework

The first step in developing an index is to develop a theoretical framework. The theoretical framework of community health determinants was constructed by referring to the models presented as health determinants in previous studies [5,6]. We then set the domains and listed indicators by referring to health-related determinants suggested by previous study in Korea.

#### Selection of indicators

We conducted a Delphi survey with 12 public health experts on April 10^th^, 2019. Indicators were evaluated on the criteria of relevance, accuracy, credibility, timeliness, accessibility, interpretability, and coherence by using a five-point Likert scale. The reliability of all questions was confirmed using the test-retest method in the pilot study conducted two weeks before the final survey. The public health experts also suggested additional indicators to be included and provided their opinions and advice.

#### Calculation of the K-CHDI

To impute the missing data, the study replaced them with the data of the previous year. Data for one indicator—educational attainment—was taken from another data source, which was 20% of the sample of the census conducted every five years. We replaced educational attainment data with those from the previous year.

The calculation of the composite index is a process of combining heterogeneous indicators of various dimensions. It requires standardization (or normalization) of the indicators before combining. We used the Min-Max normalization method, which prior studies also used, as well as integrated index calculation.

For weighting the indicators, we applied a subjective approach in the Delphi surveys of experts. An analytic hierarchy process (AHP) was performed by 18 health experts to measure the relative weights of domain and indicators of each health determinant. A nine-point scale was used in the AHP for making pairwise comparison, and the consistency index was calculated for logical consistency.

The composite index was calculated by multiplying the standardized indicator value by the weight of the domain and the weight of the indicator. The index has a value between 0 and 1, and the closer to 1, the community has healthier determinants.

$$I_{ij}=\sum x_{ij}\times \;w_{i}\times \;w_{j}$$

I_ij_: Community Health Determinants Index

x_ij_: Standardized value of Domain i, indicator j

w_i_: weight of Domain (i)s

w_j_: weight of indicator (j)

### Validity of the K-CHDI

#### Analysis estimating the association between K-CHDI and health outcomes

Disability-adjusted life years (DALYs) are the sum of the years of life lost (YLLs) and years lived with the disability (YLDs). Scatter plots correlating K-CHDI with mortality, YLLs, YLDs, and DALYs were graphed. We correlated health outcomes with the K-CHDI to confirm the validity using Pearson’s correlation analysis. The Pearson correlation coefficient was interpreted as significant or not based on the associated p-value. We also compared the coefficients between the K-CHDI and health outcomes with the deprivation index and health outcomes.

#### Health outcomes

The study estimated health outcomes by using mortality rate and DALYs. Mortality rate per 100,000 population was calculated by subnational-, sex-, and age-specific death rate, obtained from Statistics Korea. YLLs were estimated by subtracting age at premature death from the life expectancy. The standard life expectancy by age and sex of the Korean population published by Statistics Korea was used. Cause of death statistics were obtained to measure the disease-specific mortality rate by age and sex, and to adjust the garbage code, we redistributed cause of death according to the algorithm developed in a previous study [7]. YLDs were calculated by measuring the incidence of disease from the Korean National Health Insurance Service data, a representative medical use database covering approximately 98% of the population. We adjusted the disability weight for each cause, obtained from the survey of the national population [8].

To standardize health outcome metrics by the regional population structure, we used the direct age standardization method with the mid-year population of Korea in 2005.

## Results

### Development of K-CHDI

We relied on a framework developed by the South East Public Health Observatory for a health poverty index [9] and structural determinants, defined by Solar and Irwin [10] as socioeconomic and political context and socioeconomic position.

**Fig 1:**
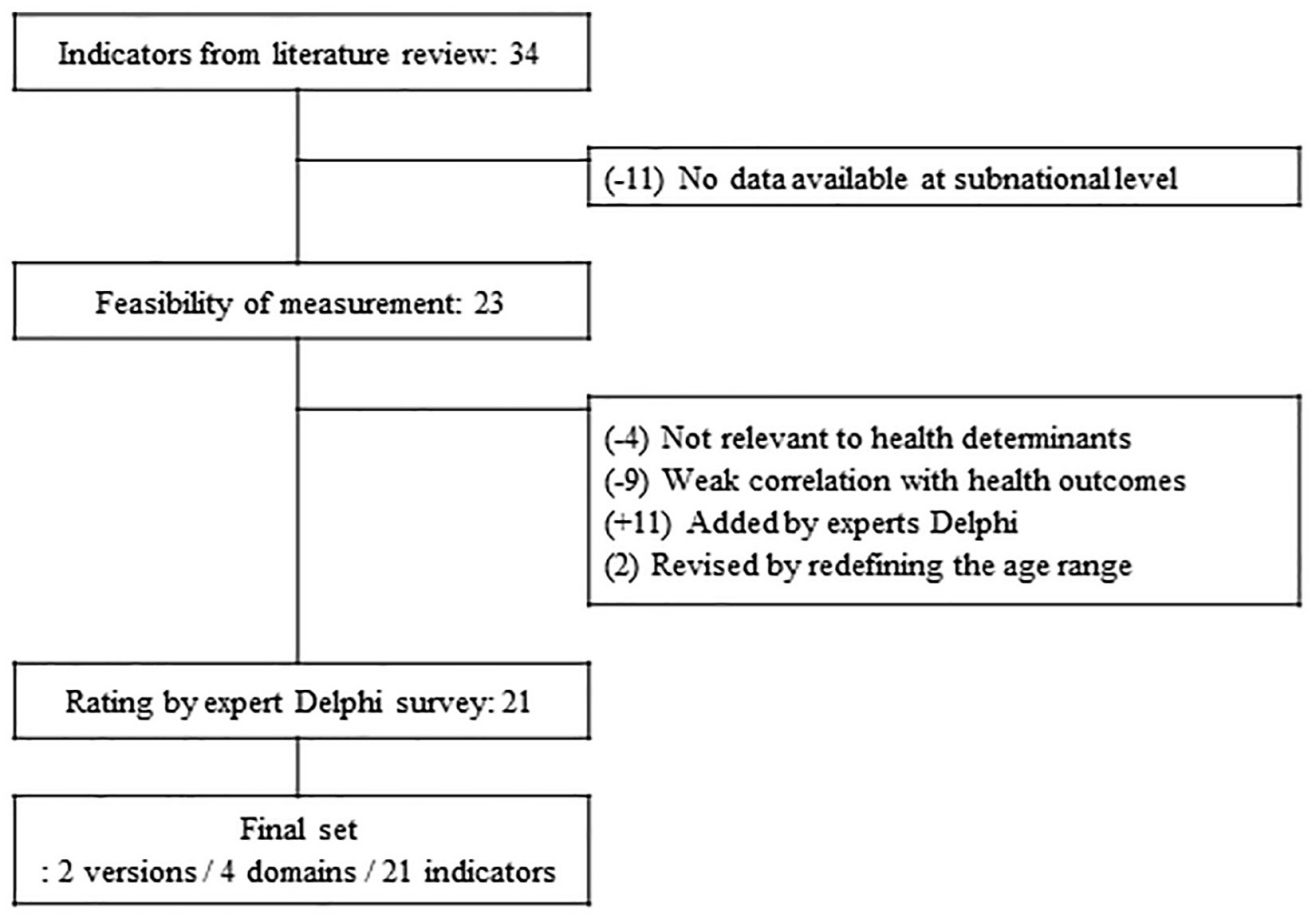
Process for the selection of K-CHDI indicator.

The indicator selection of Korean subnational health determinants was guided by the interplay of theoretical considerations and the Delphi survey. Based on a review of the literature evaluating health determinants in Korea, our initial strategy identified 34 indicators. Of these, 11 were excluded for not having official and reliable data at the subnational level. The selection of indicators was also informed by public health experts’ opinion; we excluded 13 indicators that scored two out of five or less in importance, relevance, and interpretability. In the opinion of our experts, health behaviors of the region should be included as the health determinants, and we categorized those indicators as an independent domain. The Delphi survey also assessed whether indicators were classified into appropriate domains: social and economic, population, resource, and health behavior. A flow diagram outlining the selection strategy is shown in Fig 1. Our 21 selected indicators are described in Table 1, with each indicator given a definition, measurement unit, and data source.

**Table 1:** Definitions of the K-CHDI indicators.

Domain	Indicator	Definition		Unit		Data source, Year
Social and Economic	Household income per capita	N	The total gross household monthly income		10,000 KRW	(KCDC) Community Health Survey, 2016
		D	Square root of the number of household members			
	Basic livelihood security household	N	The number of basic livelihood security households	×100	%	(KCDC) Community Health Survey, 2016
		D	Total number of households			
	Employment rate (15-64y)	N	The number of employed persons	×100	%	(KCDC) Community Health Survey, 2016
		D	Population aged 15 to 65			
	Educational attainment (≥15y)	N	Average years of education received		Year	(Statistics Korea) Census-20% Sample, 2015
		D	Population aged 15 and older			
Population	Total fertility rate (15-25y)	N	Age-specific total fertility rate		N	(Statistics Korea) Census, 2016
		D	The number of years in each age group			
	Sex ratio (Female/Male)	N	Male population	×100	%	(Ministry of the Interior and Safety) Population registration, 2016
		D	Female population			
	Elderly population	N	Population aged 65 and older	×100	%	(Statistics Korea) Census, 2016
		D	Total population			
	Elderly living alone	N	Population living alone	×100	%	(Statistics Korea) E-subnational statistics, 2016
		D	Population aged 65 and older			
	Disabled population	N	The number of disabled persons	×100	%	(National Health Insurance Service) Long Term Care Insurance Statistical Yearbook, 2016
		D	Total population			
	Urban population	N	Population living in urban area	×100	%	(Korea Land and Housing Corporation) Statistics of urban planning, 2016
		D	Total population			
Resource	Number of hospitals (per 1000)	N	The number of hospitals	×1000	N	(Statistics Korea) E-subnational statistics, 2016
		D	Total population			
	Number of doctors (per 1000)	N	The number of doctors	×1000	N	(Statistics Korea) E-subnational statistics, 2016
		D	Total population			
	Health expenditure proportion	N	Annual expenditure on health	×100	%	(Statistics Korea) E-subnational statistics, 2016
		D	Total annual expenditure			
	Financial independence	N	Annual revenue from community-own	×100	%	(Statistics Korea) E-subnational statistics, 2016
		D	Total annual revenue			
	Unmet treatment need rate	N	Respondents who had an unmet need for a medical treatment during the past year	×100	%	(KCDC) Community Health Survey, 2016
		D	Total respondents			
Health Behavior	Smoking rate	N	Respondents who smoke “every day” or “often”	×100	%	(CDC) Community Health Survey, 2016
		D	Respondents who had smoked 100 cigarettes in their lifetime			
	High-risk drinking rate	N	Respondents who drank more than twice a week (7 glasses for men or 5 glasses for women per day)	×100	%	(KCDC) Community Health Survey, 2016
		D	Respondents who drank at least once during the past year			
	Obesity rate	N	Respondents whose total BMI was over 25	×100	%	(KCDC) Community Health Survey, 2016
		D	Total respondents			
	Low salt diet rate	N	Respondents who answered “yes” to one of the three low-salt related questions: 1) Eat low salt, 2) No added salt or soy sauce on food, 3) No dipping sauce when eating fried food	×100	%	(KCDC) Community Health Survey, 2016
		D	Total respondents			
	Physical activity rate	N	Respondents who had intensive physical activity over 20 minutes a day or moderate physical activity over 30 minutes per day for at least five days a week in the last week	×100	%	(KCDC) Community Health Survey, 2016
		D	Total respondents			
	Health screening rate	N	Respondents who had health examinations during the past two years	×100	%	(KCDC) Community Health Survey, 2016
		D	Total respondents			

N = numerator; D = denominator; KCDC = Korea Centers for Disease Control & Prevention; KRW = Korean Won.

The raw data of the 21 indicators were standardized using the Min-Max method to avoid adding up variables with different measurement units. When rescaling, we chose the “direction” of each indicator, that is, where health improves or decreases with the indicator values. Values ranged from 0 to 1, with 1 being the most beneficial for health.

We applied weighting for the indicators and domains from the AHP. The consistency of answers was 83.3%. Out of the four domains, social and economic was the most important (0.518), followed by population (0.304), health behavior (0.107), and resources (0.071). In the social and economic domain, household income per capita (0.429) showed the highest weight, followed by basic livelihood security households (0.244), educational attainment (0.206), and employment rate (0.121). The elderly population rate (0.246) in the population domain, unmet treatment need rate (0.087) in the resource domain, and smoking rate (0.302) in the health behavior domain were estimated as the highest weighting in each domain. The weights for indicators are described in the Appendix, Table 1.

The values of the K-CHDI for each subnational region were measured by multiplying the standardized indicator value by the weight of the domain and the weight of the indicator.

### K-CHDI and health outcomes

To examine the validity of integrating the K-CHDI’s indicators into one domain, we conducted correlation analysis between the individual indicators and domain’s index. In the social and economic domain, household income per capita showed the strongest correlation (r = 0.961, p<.0001), followed by average education years (r = 0.917, p<.0001) and basic living security household (r = 0.824, p<.0001). Employment rate (r = 0.478) had relatively week correlation but was nevertheless statistically significant (p<.0001). In the domain of population, all indicators except sex ratio (r = 0.091, p.152) had significant correlation with the domain’s index. In the resource domain, the coefficient was relatively lower than those of the other domains (r = 0.529 ~ 0.675); however, all indicators had statistically significant correlation with the domain’s index (p<.0001). In the health behavior domain, the smoking rate (r = 0.861, p<.0001) was the strongest correlation, and the physical activity rate had non-statistically significant correlation (r = 0.273, p = .630).

**Fig 2:**
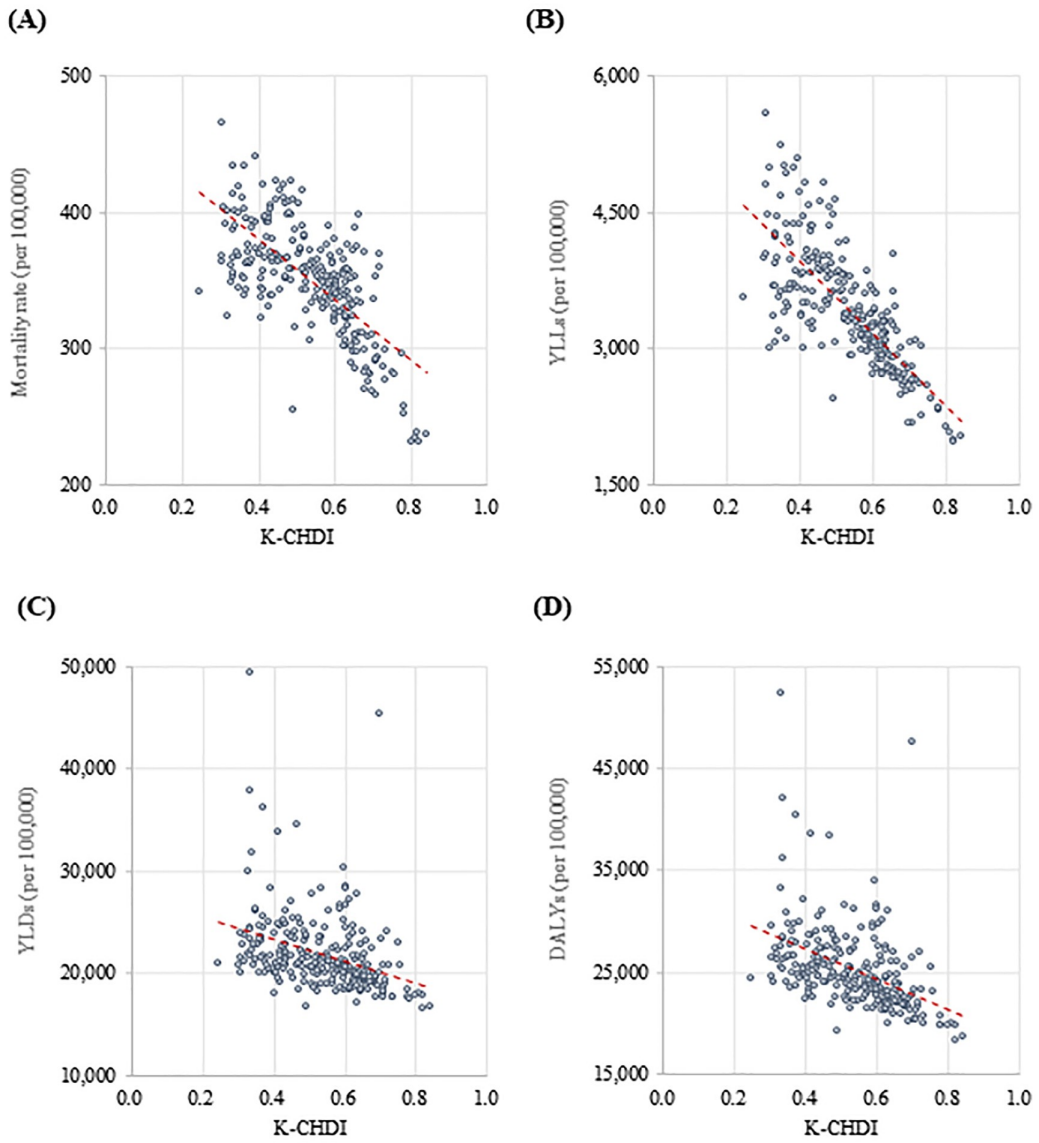
Correlation between K-CHDI and health outcomes. (A) Mortality rate, (B) YLL, (C) YLD, (D) DALY. K-CHDI = Korean Community Health Determinants Index; YLLs = years of life lost; YLDs = years lived with disability; DALYs = disability adjusted life years.

As a result of the correlation analysis between the K-CHDI and health outcome metrics for 250 regions, the coefficient was significant in all health outcomes: mortality, YLLs, YLDs, and DALYs. The greater the K-CHDI, the less the disease burden. For the K-CHDI, the correlation coefficient was greatest in YLL (r = -0.770, p<.0001), followed by mortality rate (r = -0.690, p<.0001), YLD (r = -0.352, p<.0001), and DALY (r = -0.455, p<.0001) (Table 2, Fig 2).

**Table 2:** Correlation coefficient between K-CHDI and health outcomes.

Index	Health Outcomes			
	Mortality rate	YLLs	YLDs	DALYs
K-CHDI	-0.690	-0.770	-0.352	-0.455
	(<.0001)	(<.0001)	(<.0001)	(<.0001)

Unit: Pearson correlation coefficient r (p-value).

K-CHDI = Korean Community Health Determinants Index; YLLs = years of life lost; YLDs = years lived with disability; DALYs = disability adjusted life years.

The correlation between the K-CHDI and mortality was the strongest in the socio-economic domain (r = -0.641, p<.0001), followed by the population domain (r = -0.639, p<.0001), resources domain (r = -0.402, p<.0001), and health behavior domain (r = -0.402, p<.0001).

The correlation between K-CHDI and health outcomes in this study (r = -0.690, p<.0001) was greater than the coefficients reported in the previous studies of the health determinants index and health outcomes. In Shin at al. [11], the regional deprivation index was correlated with mortality rate with r = 0.316(p<.0001), while the health determinants index was correlated with self-reported health with r = 0.476 (p<.01).

**Fig 3:**
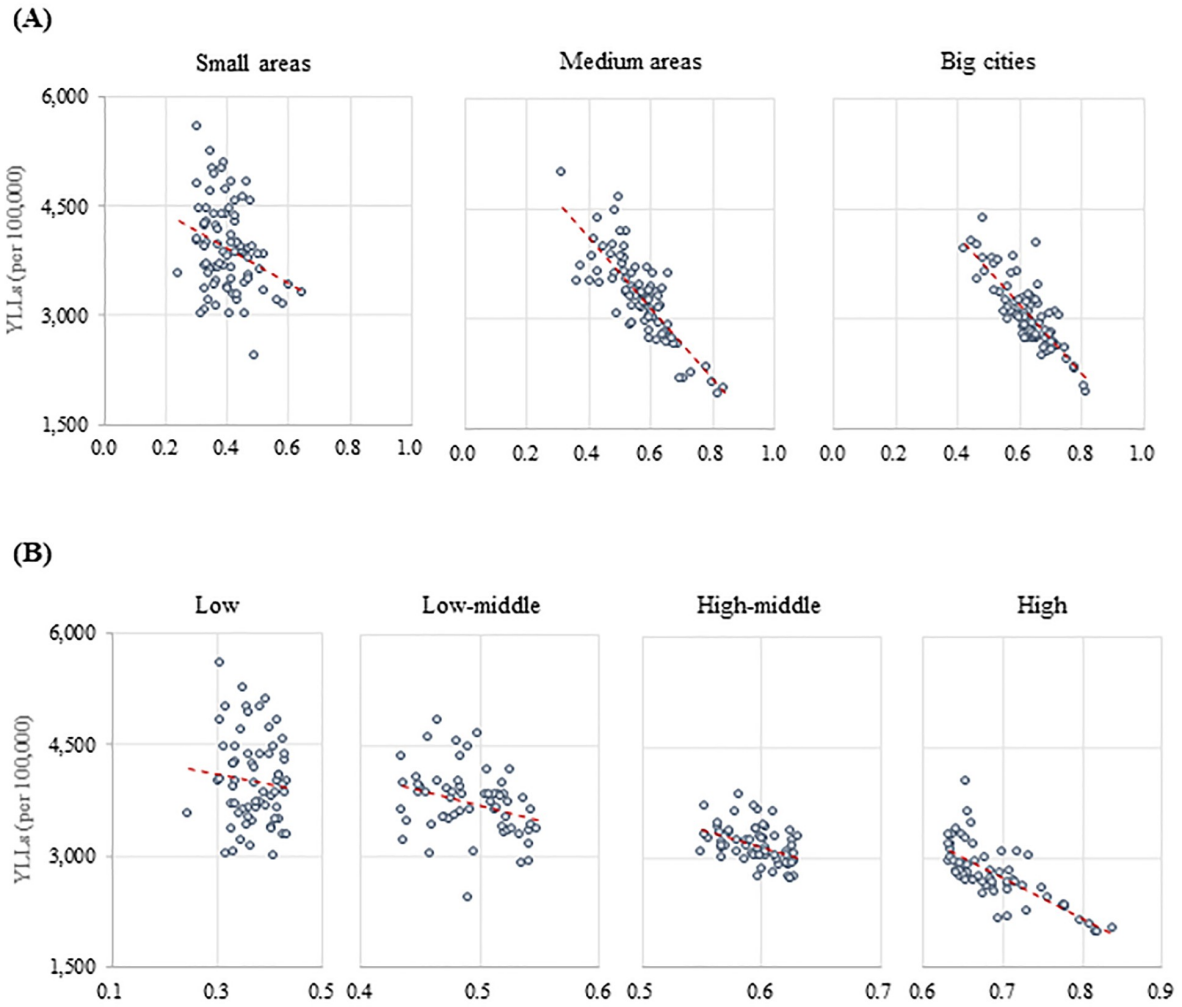
Correlation between K-CHDI and YLLs by subgroups. (A) Regions, (B) K-CHDI-quartiles. K-CHDI = Korean Community Health Determinants Index; YLLs = years of life lost; YLLs.

Fig 3 illustrates the correlations between the K-CHDI and the YLL by subgroups. We classified the 250 regions by population size as follows: large cities, if they had a population of over one million; medium-sized areas, if the population of the “Si” and “Gu” combined comprised less than one million; small areas for the other “Gun”. The correlation between the K-CHDI and the YLL was stronger in large cities (r = -0.815) than medium-sized areas (r = -0.786), and small areas showed the lowest correlation (r = -0.224) (Fig 3A). The 250 regions could be grouped by the K-CHDI quartiles: high, high-middle, low-middle, and low. According to the K-CHDI quartile groups (Fig 3B), the correlation between the K-CHDI and the YLL was stronger in the high group (r = -0.647) than in the low group (r = -0.008).

## Discussion

### Development of the K-CHDI

K-CHDI was developed by standardizing the Min-Max normalization method and applying the weights calculated by the public health experts’ AHP. The index consists of four domains and 21 indicators, ranging from 0 to 1; closer to 1 means that the health determinants in the community tend in a positive direction.

We chose a composite index as being easier to interpret than individual indicators. There is controversy over the selection of indicators, it is possible to summarize complex and multidimensional realities to support decision making, instead of being limited in interpreting the indicator itself, although there is controversy over the selection of indicators [12]. In a previous study that developed regional or national health-related indices [11,13], different indicators were selected, and calculation methods were different. Our study was based on social determinants of health and indicators selected by a Delphi survey to guarantee transparency while considering the available data. The experts who participated in the survey evaluated whether the indicator met the objectives of the index, was measurable, and could represent its characteristics. In addition, the correlation analysis between the suggested indicators and the health outcomes supported their evaluation. Those indicators having a weak explanation of disease burden which were nonetheless commonly included in the previous study to measure health determinants were reviewed for relevance. For example, in the case of the number of cars registered per person, the correlation with health was not statistically significant, which was inadequate for measuring income in rural areas, where traveling distances takes longer with poor public transportation. Automobiles are considered a necessity, not an asset, for farming and living [11]. In addition, in rural areas where most people own their homes, housing does not represent income level, in contrast to city residents [14]. We did not unconditionally select indicators suggested in previous studies and did not unconditionally exclude them even if the correlation with health outcomes was not significant. An index consisting only of indicators highly correlated with health outcomes can improve validity; however, the prior objective of K-CHDI was to represent Korean community health determinants rather than develop a highly validated index. For that reason, we used the correlation between the individual indicators and health outcomes as the reference when choosing a specific indicator out of many with similar characteristics.

### Utilization of the K-CHDI

To verify the validity of the K-CHDI, we analyzed the correlation between indicators and domain index, and domain index and total aggregated index. The coefficient was statistically significant; therefore, it is reasonable to develop and use indicators in each domain as one composite indicator.

Since the index is calculated by integrating a number of indicators, it is limited in interpreting which indicators have a greater effect and are more important. However, out of all the indicators, income per capita demonstrated the strongest relationship with health outcomes, which was in accordance with the result of the domain weight. The indicators of the resource domain showed a relatively low correlation with health outcomes. These results were consistent with a previous study conducted in Korea [15]. The correlation coefficient between the resources including the number of beds and doctors, and mortality rate were relatively lower than other indicators. A study outside Korea also reported a low correlation between health outcomes and resources, which was lower than environmental factors and housing [16]. In addition to the number of beds or doctors, further research on identifying other resource indicators affecting patients’ healthcare infrastructure accessibility in the community is needed. However, no study to date has reported healthcare resources as health determinants in Korea.

To determine whether K-CHDI explains health outcomes at the subnational level in Korea, we also analyzed the correlations between 250 subnational K-CHDI and health outcomes. The statistically significant correlation between K-CHDI and disease burden implies that the K-CHDI is a useful proxy for health outcomes at the subnational level. The correlation between K-CHDI and health outcomes in this study was greater than the coefficients reported in previous studies of the health determinants index and health outcomes.

K-CHDI described YLLs that indicate premature death better than those that indicate mortality. This implies that the burden of disease due to premature death is greater than mortality itself depending on health determinants. However, it correlated relatively weakly with YLD and DALY, which represented disability. Although there were no previous studies analyzing the health determinants of disease burden with YLL and YLD, Kim [12] showed that there were significant correlations between health determinants and self-rated health; however, the correlation coefficient was less than 0.5, indicating that the ranking of health determinants could not be interpreted as a ranking of health outcomes. This is because the health determinants affect mortality, and morbidity will be different.

K-CHDI is more useful for evaluating health status than the regional deprivation index, which was previously used as a determinant of health at the subnational level. In Shin et al. [11], which developed the regional deprivation index, educational status was excluded because it could not represented the deprivation of the community. Son [17] also reported that educational and occupational status were not correlated with deprivation variables at the regional level. This implies that the indices of regional deprivation and health determinants should be constructed differently according to the purpose of the development. K-CHDI is useful for analyzing and evaluating subnational health outcomes and will prove a valuable standard for comparison.

This study has some limitations. To select the indicators and measure the relative weights of the domains and indicators, the study conducted a Delphi survey with 12 and 18 public health experts, respectively. Although there are no specific guidelines regarding the number of experts included, and it varies from 10 to 50 in previous studies, a large number of experts can change the priorities of the indicators. In addition, this study is a cross-sectional study, and the results are limited to the study period, and also limited in verifying the temporality between the index and health outcomes. Further studies with longitudinal data can improve the use of the index for predicting health outcomes. The GBD study [2] used a sociodemographic index as a basis for predicting health outcomes such as mortality, life expectancy, and disease burden. Life expectancy at birth is rising; however, the difference between high and low socioeconomic status countries has increased. Monitoring health outcomes according to sociodemographic level is necessary for national and subnational health policy.

We propose using K-CHDI as a standard for assessing and comparing health outcomes at the subnational level in Korea. Regions having similar status in health determinants can be classified according to the K-CHDI quartile. For now, comparisons of subnational health are based on geographical proximity. However, K-CHDI allows for reasonable interpretation by comparing regions with similar structural characteristics. Even in regions with the same index, the indicators that make up the index may be different, which may raise the question of whether it is reasonable to view the regions as similar. Nevertheless, the K-CHDI represents a reference standard for estimating health status using health determinants as composite indicators at the subnational level. Considering the SDI’s use is limited to factors that explain current socioeconomic status of a given country or health outcomes, an index that includes health indicators should be developed to better explain the health differences among certain country. The findings of this study can be adapted and used in other settings, in particular with similar health determinants, if there is a regional data source. In addition, the indicators, which the index is composed of, could be replaced with prior indicators in each country.

## Supporting information

S1 Table Weights for domains and indicators by analytic hierarchical process. (DOCX)
