# Supplementary material for: Development of the Korean Community Health Determinants Index (K-CHDI)
Source: PLoS One. 2020 Oct 8;15(10):e0240304. doi: 10.1371/journal.pone.0240304 (PMC7544091; doi:10.1371/journal.pone.0240304)
Supplement: S1 Table — (DOCX) [file pone.0240304.s001.docx]

**S1 Table. Weights for domains and indicators by analytic hierarchical process**

| Domain | | Indicator | | Total (Ranking) | |
| --- | --- | --- | --- | --- | --- |
| Social and  economic | .419 | Household income per capita | .429 | .180 | (1) |
|  |  | Basic livelihood security household | .244 | .102 | (2) |
|  |  | Employment rate (15-64y) | .121 | .051 | (7) |
|  |  | Educational attainment (≥15y) | .206 | .086 | (3) |
| Population | .249 | Fertility rate (15-25y) | .205 | .051 | (7) |
|  |  | Sex ratio (Female/Male) | .246 | .061 | (5) |
|  |  | Elderly population | .196 | .049 | (9) |
|  |  | Elderly living alone | .143 | .036 | (12) |
|  |  | Disabled population | .110 | .027 | (13) |
|  |  | Urban population | .100 | .025 | (14) |
| Resource | .073 | Number of hospitals (per 1000) | .245 | .018 | (18) |
|  |  | Number of doctors (per 1000) | .192 | .014 | (20) |
|  |  | Independent rate of finance | .148 | .011 | (21) |
|  |  | Health expenditure proportion | .328 | .024 | (15) |
|  |  | Unmet treatment need rate | .087 | .022 | (16) |
| Health behaviour | .259 | Smoking rate | .302 | .078 | (4) |
|  |  | High-risk drinking rate | .225 | .058 | (6) |
|  |  | Obesity rate | .167 | .043 | (10) |
|  |  | Low salt diet rate | .065 | .017 | (19) |
|  |  | Physical activity rate | .163 | .042 | (11) |
|  |  | Health screening rate | .077 | .020 | (17) |
